# Dietary folate intake and metabolic dysfunction-associated steatotic liver disease: a prospective cohort study

**DOI:** 10.1186/s12986-026-01141-0

**Published:** 2026-05-20

**Authors:** Qingchun Li, Xinyu Zhang, Fengyuan Zhao, Dongfeng Zhang, Weijing Wang, Zhongyang Zhang, Feng Yang

**Affiliations:** 1 Department of Epidemiology and Health Statistics, School of Public Health, Qingdao University, Qingdao, China; 2 Qingdao International Travel Healthcare Center, Qingdao Customs District P. R. China, Qingdao, China; 3 Lianyungang Hospital of Traditional Chinese Medicine, Lianyungang City, Jiangsu Province China; 4 Institute of Immunization Planning, Qingdao Municipal Center for Disease Control and Prevention, Qingdao, China

**Keywords:** Dietary folate intake, MASLD, Cohort Study, Inflammation, Mediation analysis

## Abstract

**Background:**

Metabolic dysfunction-associated steatotic liver disease (MASLD) is a prevalent chronic disease, but it remains unclear whether it is related to dietary folate. This research sought to investigate the association between dietary folate and MASLD.

**Methods:**

This cohort study utilized the UK Biobank (UKB) database (*N* = 58,047). Dietary folate intake was assessed using an online dietary questionnaire, and MASLD was ascertained through International Classification of Diseases, Tenth Revision (ICD-10). Cox proportional hazard regression, mediation analysis and restricted cubic splines (RCS) were employed to investigate the association and dose-response relationship between dietary and MASLD. The stability of findings was verified by stratified analyses and sensitivity analyses.

**Results:**

Dietary folate intake was observed to be negatively associated with MASLD in cohort studies. During a median follow-up of 12.18 years, 691 cases of MASLD occurred. The risk of MASLD decreased by 24% (HR = 0.76, 95% CI: 0.59–0.97) among the participants in the highest dietary folate intake relative to lowest quartile. The protective effect was more pronounced in participants aged < 60 years (HR = 0.63, 95% CI: 0.45–0.89), male (HR = 0.63, 95% CI: 0.44–0.89), and obese participants (HR = 0.68, 95% CI: 0.48–0.95). The research results remained robust in the sensitivity analyses that excluded participants with ≤ 2 years of follow-up time, extremely folate intake, and other conditions. C-reactive protein (CRP) explained 6.56% of the association between dietary folate and MASLD. The dietary folate intake had an L-shaped relationship with the risk of MASLD (*P for nonlinearity* = 0.003).

**Conclusions:**

Moderate folate intake (approximately 300 µg) may reduce the risk of MASLD, suggesting that promoting folate-rich diets may be a viable and cost-effective strategy for MASLD prevention.

**Supplementary Information:**

The online version contains supplementary material available at 10.1186/s12986-026-01141-0.

## Background

Metabolic dysfunction-associated steatotic liver disease (MASLD) is widely recognized as a major cause of chronic liver disease, afflicting over 30% of adults [1, 2]. MASLD is defined as the simultaneous presence of hepatic steatosis and at least one specific cardiometabolic risk factor [3, 4]. MASLD has a wide range of clinical manifestations, it could develop to liver fibrosis and cirrhosis, and also elevates the risk of cardiovascular events, chronic kidney diseases, and extrahepatic malignancies [5, 6]. Beyond these, MASLD is also associated with a decrease in quality of life and brings a substantial socioeconomic burden [7, 8]. The pathophysiology of MASLD may involve complex interactions among insulin resistance, oxidative stress, lipid dysmetabolism, and lifestyle factors such as high-sugar and high-protein intake, but the specific mechanism remains unclear [9–11]. Given the current lack of effective pharmacological therapies, lifestyle interventions, especially dietary adjustment has been emphasized as a key management strategy for MASLD [12].

In recent years, the role of nutrients in the occurrence and development of MASLD has increasingly received attention. Dietary fatty acids, vitamins (C, E, etc.), and minerals (manganese, selenium, etc.) were all associated with the risk of MASLD [13–18]. As a pivotal water-soluble B vitamin, folate functions as an important cofactor in one-carbon metabolism, and is essential for nucleic acid biosynthesis, amino acid metabolism, and immune responses [19, 20]. Folate deficiency could promote the release of pro-inflammatory factors, destroy lipid metabolism, and ultimately develop hepatic steatosis and fibrosis [21, 22]. These evidences suggested a plausible hypothesis that dietary folate intake could be linked to MASLD.

Previous studies on folate were conducted in serum and red blood cells (RBC), suggesting that MASLD was negatively correlated with serum folate levels while positively correlated with RBC folate [9, 23]. Additionally, previous studies have also explored the association between folate and non-alcoholic fatty liver disease (NAFLD), indicating that dietary folate and serum folate were inversely associated with NAFLD [24–27], which provided a critical theoretical basis for exploring the relationship between MASLD and folate.

However, there was no study linking dietary folate to MASLD and exploring the dose-response relationship between them. Therefore, to probe the association between dietary folate intake with MASLD, we performed cohort analyses leveraging UK Biobank (UKB) data. Furthermore, the possible mechanism was explored through mediation analysis, and the dose-response relationship was also investigated using restricted cubic splines (RCS).

## Methods

### Study population and design

This research was based on UKB, a large-scale, population-based prospective cohort. From 2006 to 2010, the UKB recruited over 500,000 participants from 22 assessment centers [28]. Comprehensive information encompassing sociodemographic, health behaviors, and medical history were collected via touchscreen surveys and direct interviews. Physical examination, along with the collection of blood and urine samples were all conducted by professionals in accordance with standardized protocols [29]. Besides, UKB bridged the national database to follow the disease conditions of participants. This study has passed the ethical review from North West Multi-Centre Research Ethics Committee (reference number: 21/NW/0157) and every participant signed an informed consent.

**Fig. 1:**
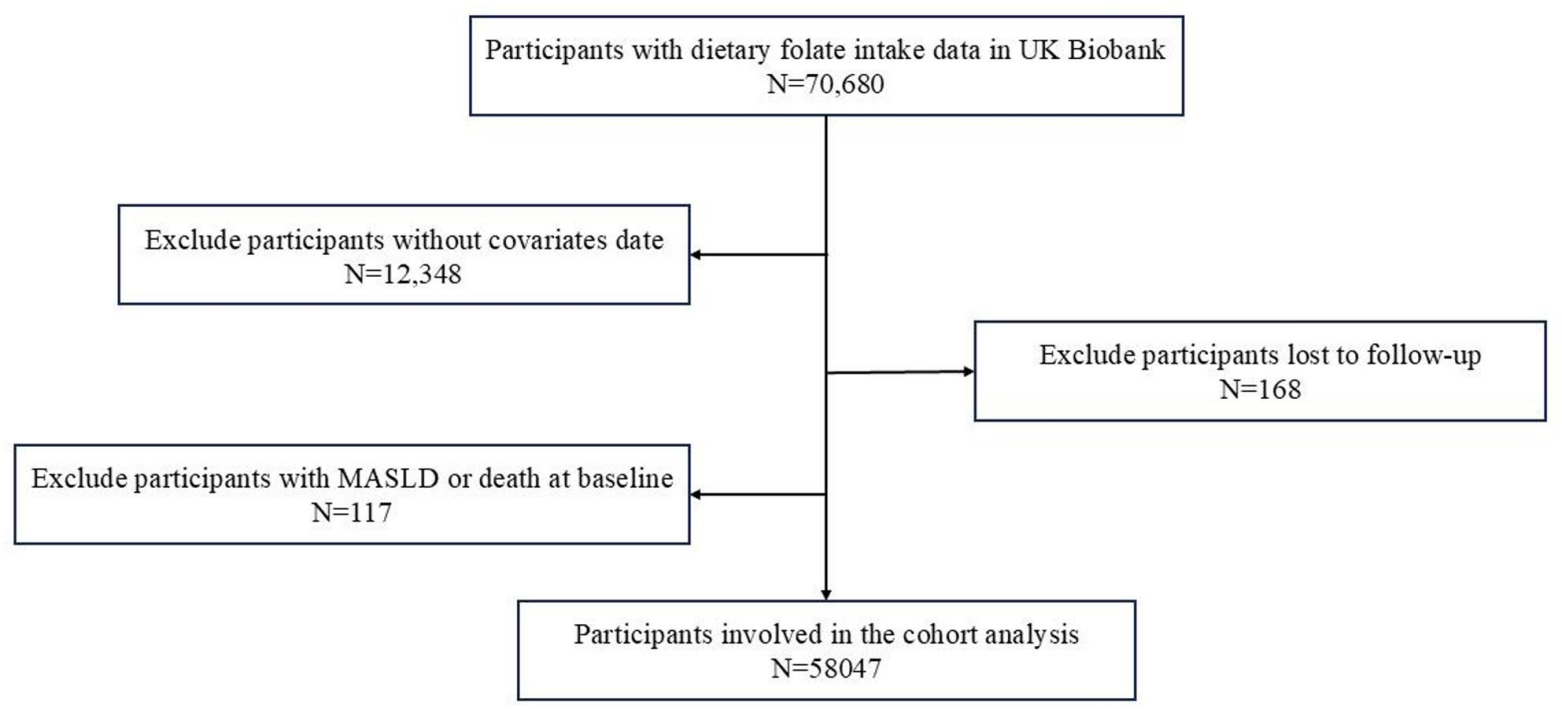
Flowchart of inclusion and exclusion

In this study, a cohort study was conducted based on UKB database. We excluded participants without covariates information and lost to follow-up, and participants with MASLD diagnosis or death during the baseline period (April 2009 to September 2010). The final cohort included 58,047 participants, and the detailed selection flowchart was shown in Fig. 1.

### Exposure

Dietary folate data were collected at baseline (from April 2009 to September 2010) via the an online 24-hour dietary questionnaire on Oxford WebQ, which could capture information about intake of 206 food and 32 beverages [30, 31]. Nutrient intakes (including dietary folate) were estimated through multiplying the intake of each food item by its nutrient content per serving and then summing these values across all items [32]. We used the dietary folate intake measured from April 2009 to September 2010 as the exposure variable (Supplementary Table S1). For analytical purposes, participants’ baseline dietary folate intake was categorized into quartiles (Q1- Q4).

### Outcome

The outcome of this research was incidence of MASLD. It was diagnosed according to the codes K75.8 and K76.0 in International Classification of Diseases, Tenth Revision (ICD-10), which aligned with consensus recommendations [33, 34]. Follow-up time began from the date of enrollment and until the first appearance of the following events: MASLD, death, withdrawal, loss to follow-up, and the culmination of the follow-up duration (December 6th, 2022).

### Covariates

We selected the following variables as covariates according to previous evidence: (1) Demographic features: age, sex, ethnicity, body mass index (BMI), educational level, and Townsend Deprivation Index (TDI). (2) Lifestyle: physical activity, energy intake, smoking status, and alcohol status. (3) Chronic diseases: hypertension and diabetes [35, 36].

### Mediator

We employed a mediation analysis to explore the mechanisms underlying the association between dietary folate intake with MASLD. Chronic inflammation is regarded as a significant contributing element in the pathogenesis and progression of MASLD [37]. C-reactive protein (CRP) is a classical systemic inflammatory biomarker, and has been shown to be positively associated with MASLD [38, 39]. Furthermore, prior evidence indicates that folate may reduce the levels of inflammation and CRP [40, 41]. Therefore, we hypothesized that the association between dietary folate intake with MASLD might be mediated by CRP and performed mediation analysis with CRP as mediator variable to verify this hypothesis. We performed a logarithmic transformation on CRP to make it follow a normal distribution or be close to a normal distribution, thereby reducing the influence of extreme values.

### Statistical analysis

Demographic characteristics were presented for the total study population and stratified by incident MASLD status. The Kolmogorov-Smirnov test was adopted to evaluated the normality of continuous variables. Since the distributions of all the continuous variables in this study were non-normal, they were displayed as median (interquartile range [IQR]). Frequencies (n) and percentages (%) were adopted to display categorical variables. We adopted the Mann-Whitney *U* test and the chi-squared (*χ²*) test for continuous and categorical variables respective to compare group differences.

To control the potential confounding factors, we constructed four sequential adjustment models: Model 1 was a crude model without any covariates. Model 2 made adjustments for age, sex, ethnicity, BMI, educational level and TDI. Based on Model 2, Model 3 additionally took into account lifestyle factors such as physical activity, energy intake, smoking and drinking status. The fully adjusted Model 4 further adjusted chronic diseases (hypertension and diabetes) from Model 3.

For the cohort study, we estimated the longitudinal association between dietary folate intake with MASLD through the Cox proportional hazards regression model. The results were described as Hazard ratio (*HR*) and 95% confidence interval (*CI*). We adopted the Schoenfeld residual test to examine the proportional hazard assumption. The concordance index (C-Index) was adopted to estimate the discriminability of the model. The range for values of C-index from 0.5 to 1.0, the closer to 1, the better prediction effect of the model [42]. The cumulative risk of MASLD across folate intake quartiles was visualized through Kaplan-Meier curves, with curve differences evaluated by the log-rank test.

The Process program in SPSS was used for a mediation analysis to probe the latent role of CRP in the association between dietary folate intake with MASLD. The Bootstrap method was applied for 5000 iterations and adjusted for all covariates included in Model 4.

To estimate the dose-response relationship of dietary folate intake and MASLD, we employed RCS. We also performed stratified analyses based on age (< 60 years, ≥ 60 years), sex (male, female), and BMI (< 25 kg/m^2^, 25–30 kg/m² and ≥ 30 kg/m²) to probe the association between dietary folate intake with MASLD among different populations.

To confirm the reliability of cohort study results, we adopted a series of sensitivity analyses: (1) Excluding participants with ≤ 2 years of follow-up time to mitigate possible reverse causality. (2) Participants who had extreme high or low energy intake (intake <1st percentile and > 99th percentile) were excluded. (3) We additionally removed participants with extremely dietary folate intake (intake <1st percentile and > 99th percentile). (4) Considering the possible confounding effects of folate and multivitamin supplements, we excluding participants who reported the use of folate or multivitamin supplement. (5) To control the impact of chronic diseases, we excluded participants with hypertension and diabetes. (6) To control for the confounding effects brought by other liver diseases, we excluded participants with any other known liver disease (ICD-10 codes: K70-K74, K75.0-K75.4, K75.9, K76.1-K76.9, and K77) at baseline. (7) We used waist circumference (WC) instead of BMI as a covariate to evaluate the robustness of our results. (8) To control the confounding effect of total energy intake on dietary folate intake, we adjusted folate intake for total energy using the residual method. (9) Considering the influence of intra-individual variability, attenuation bias and confounding dietary patterns, we used the average value of dietary folate obtained from multiple measurements and performed the residual method to correct based on total energy intake, and further adjusted the dietary fiber and consumption of fruits and vegetables.

All statistical analyses of this study were done with the aid of software R (version 4.5.1) and SPSS (version 24.0). A two-sided *P*-value < 0.05 was regarded to be statistically significant.

## Results

### Demographic characteristic

There were 58,047 participants in all for the cohort analysis. The participants were categorized according to MASLD status and their demographic characteristics were presented in Table 1. Participants with MASLD were characterized by an average older, a higher BMI, and a lower educational level than those without MASLD. Similarly, the prevalence rates of hypertension and diabetes were considerably elevated in the MASLD group, indicating a less favorable metabolic status. Furthermore, the MASLD group had the largest percentage of participants with the lowest folate intake (Q1) (30.7%).

**Table 1:** Demographic characteristics of participants

	Overall	Non-MASLD	MASLD	*P*
	(*N* = 58047)	(*N* = 57356)	(*N* = 691)	
Age (years, median [IQR])	57(14)	57(14)	58(13)	**0.003**
Sex (n, %)				0.269
Female	31,116(53.6)	30,760(53.6)	356(51.5)	
Male	26,931(46.4)	26,596(46.4)	335(48.5)	
Ethnicity (n, %)				0.654
White	54,536(94.0)	53,884(93.9)	652(94.4)	
Other	3511(6.0)	3472(6.1)	39(5.6)	
BMI (kg/m^2^, median [IQR])	26.39(5.61)	26.36(5.57)	30.63(7.14)	**< 0.001**
Education level (n, %)				**< 0.001**
Below the college	31,514(54.3)	31,060(54.2)	454(65.7)	
College or above	26,533(45.7)	26,296(45.8)	237(34.3)	
TDI (median [IQR])	-1.94(3.88)	-1.95(3.87)	-1.08(5.11)	**< 0.001**
Physical activity (n, %)				**< 0.001**
Low	9882(17.0)	9698(16.9)	184(26.6)	
Mid	24,113(41.5)	23,824(41.5)	289(41.8)	
High	24,052(41.4)	23,834(41.6)	218(31.5)	
Energy intake (KJ, median [IQR])	8335.24(3584.50)	8336.57(3575.93)	8219.71(4480.44)	0.097
Smoking status (n, %)				**< 0.001**
Never	32,729(56.4)	32,416(56.5)	313(45.3)	
Previous	20,496(35.3)	20,195(35.2)	301(43.6)	
Current	4822(8.3)	4745(8.3)	77(11.1)	
Alcohol status (n, %)				**< 0.001**
Never	2042(3.5)	2002(3.5)	40(5.8)	
Previous	1947(3.4)	1910(3.3)	37(5.4)	
Current	54,058(93.1)	53,444(93.2)	614(88.9)	
Hypertension (n, %)				**< 0.001**
No	42,179(72.7)	41,800(72.9)	379(54.8)	
Yes	15,868(27.3)	15,556(27.1)	312(45.2)	
Diabetes (n, %)				**< 0.001**
No	55,308(95.3)	54,738(95.4)	570(82.5)	
Yes	2739(4.7)	2618(4.6)	121(17.5)	
Dietary folate intake (n, %)				**0.003**
Q1	14,512(25.0)	14,300(25.0)	212(30.7)	
Q2	14,512(25.0)	14,348(25.0)	164(23.7)	
Q3	14,511(25.0)	14,342(25.0)	169(24.5)	
Q4	14,512(25.0)	14,366(25.0)	146(21.1)	
Dietary folate intake (ug, median [IQR])	305.45(160.31)	305.60(160.19)	292.34(164.71)	**< 0.001**

MASLD: metabolic dysfunction-associated steatotic liver disease. IQR: interquartile range; BMI: Body Mass Index; TDI: Townsend Deprivation Index; The P was in bold to indicate statistical significance.

### Cohort study

A longitudinal analysis was conducted on 58,047 participants. Over a median follow-up of 12.18 years, 691 incident cases of MASLD were documented in all. According to the Schoenfeld test, the Cox model satisfied the proportional hazards assumption, with global *P* value = 0.463 (Supplementary Figure S1). Model 1 of the Cox proportional hazards regression models (with Q1 as reference) showed that higher dietary folate intake had a negative correlation with MASLD (Table 2). After further adjustment for covariates, the associations between Q2 and Q3 and the risk of MASLD were no longer significant. In the Model 4, the risk of MASLD with highest of dietary folate intake quartile could be reduced 24% relative to lowest quartile (HR = 0.76, 95% CI: 0.59–0.97). The final model exhibited good discriminability with a C-index of 0.763 (0.745–0.780).

**Table 2:** Longitudinal association between dietary folate intake and MASLD

Folate	Participants/Case	Model 1		Model 2		Model 3		Model 4		
		HR (95%CI)	*P*	HR (95%CI)	*P*	HR (95%CI)	*P*		HR (95%CI)	*P*
Q1	14,512/212	Ref	**-**	Ref	**-**	Ref	**-**		Ref	**-**
Q2	14,512/164	0.77(0.63–0.94)	**0.012**	0.83(0.68–1.02)	0.077	0.86(0.69–1.06)	0.150		0.86(0.69–1.06)	0.150
Q3	14,511/169	0.80(0.65–0.97)	**0.027**	0.87(0.71–1.07)	0.178	0.91(0.73–1.13)	0.386		0.91(0.73–1.13)	0.392
Q4	14,512/146	0.69(0.56–0.85)	**< 0.001**	0.72(0.58–0.90)	**0.003**	0.77(0.60–0.99)	**0.038**		0.76(0.59–0.97)	**0.027**
*P* for trend		**0.001**		**0.006**		0.063			**0.046**	

HR: hazard ratio. CI: confidence interval. P: significant level. Q1: reference group. Model 1: not adjusted any covariates. Model 2: adjusted for age, sex, ethnicity, BMI, education level and TDI. Model 3: further adjusted for, physical activity, energy intake, smoking status and alcohol status. Model 4: further adjusted for hypertension and diabetes; The P was in bold to indicate statistical significance.

Kaplan-Meier curves illustrated significant differences in the cumulative risk of MASLD across dietary folate intake quartiles (log-rank *P* = 0.003) (Supplementary Figure S2). Participants in Q1 exhibited the highest cumulative risk throughout the follow-up period, whereas those in Q4 had the lowest risk of MASLD.

### Mediation analysis

**Fig. 2:**
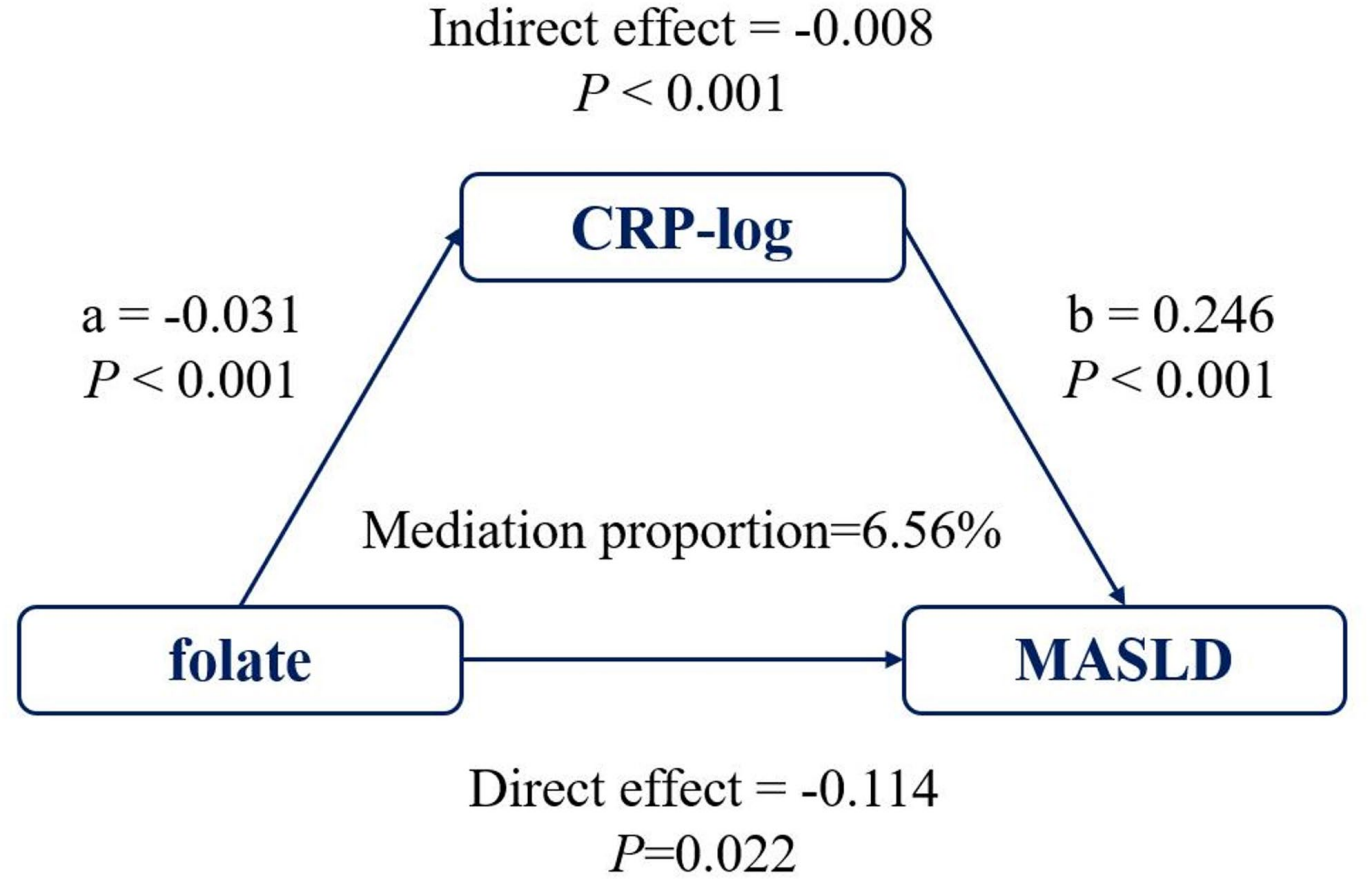
Mediation analysis path diagram. Mediation analysis of CRP on the association between dietary folate intake and MASLD. Mediation proportion= Indirect effect / [Indirect effect + Direct effect]. Adjusted for age / sex / ethnicity / BMI / education level/ Townsend deprivation index / physical activity level / energy intake / smoking status / alcohol status / hypertension / diabetes

Mediation analysis was employed with CRP as the mediating variable. According to the results of mediation analysis, CRP was found to partially mediated the association of dietary folate intake with MASLD, accounting for 6.56% of the total effect (Fig. 2). Specifically, higher dietary folate intake could reduce the CRP levels (a = -0.031, *P* < 0.001), while higher CRP levels could increase the risk of MASLD (b = 0.246, *P* < 0.001). The indirect effect mediated by CRP (a*b) was − 0.008 (*P* < 0.001) and the dietary folate had a direct effect on MASLD of -0.114 (*P* = 0.022).

### Restricted Cubic Spline

**Fig. 3:**
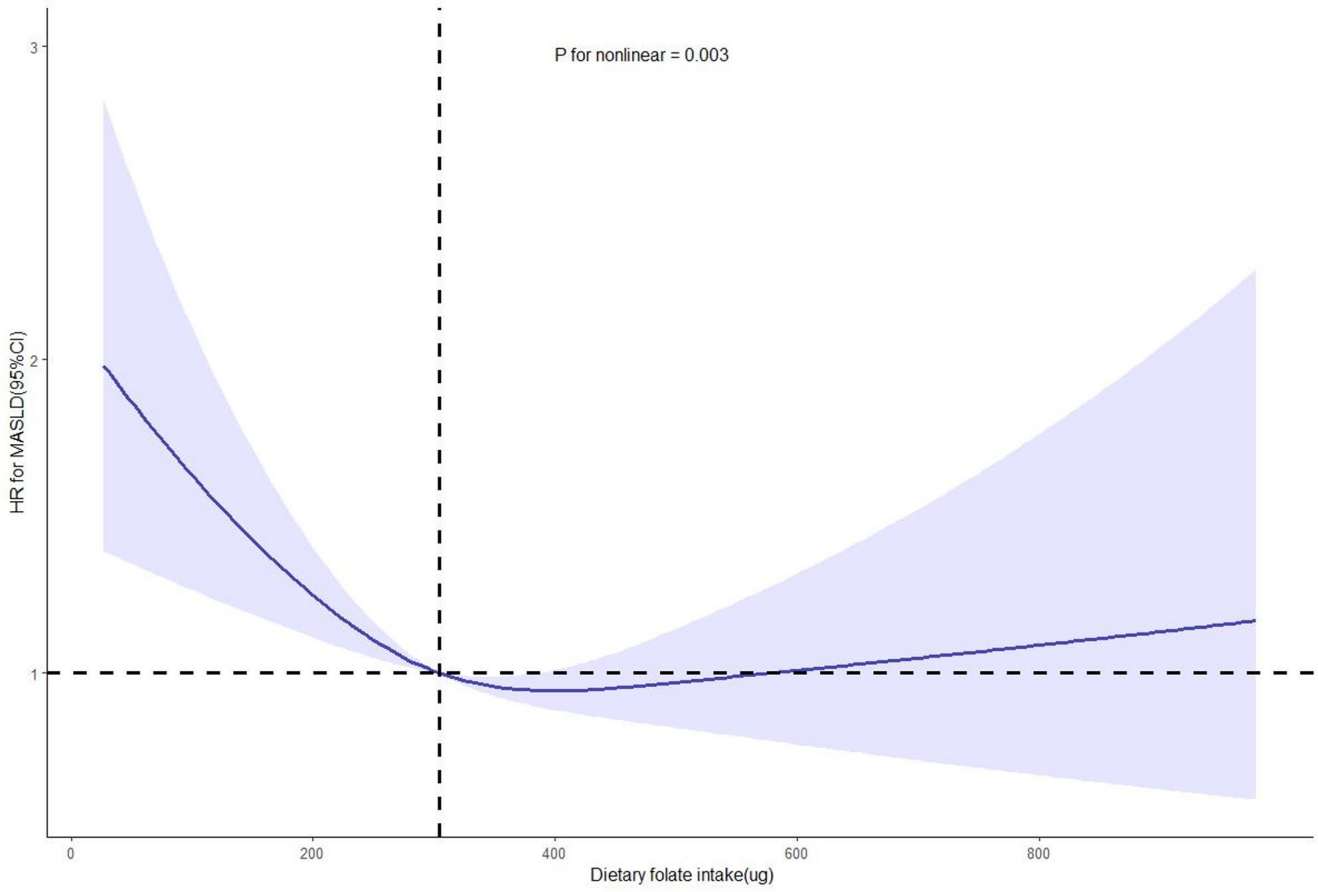
Restricted cubic spline curve of dietary folate intake and MASLD. The blue lines are HR estimates, and the light blue areas are 95%CI for HRs

Dietary folate intake was found to have a significant, nonlinear L-shaped dose-response relationship with the risk of MASLD in RCS (*P for nonlinearity* = 0.003) (Fig. 3). When folate intake was at a relatively low level (< approximately 300 µg), the risk of MASLD decreased significantly with the increase in folate intake, and the decrease of MASLD risk tended to level off when the intake exceeded approximately 300 µg.

### Stratified analyses

Table 3 showed the results of the stratified analyses. No significant interactions between dietary folate intake and age, sex, or BMI were detected (all *P for interaction* > 0.05). Nonetheless, the protective effect of folate was evident within certain subgroups. Specifically, this protective effect was existed among participants aged < 60 years (HR = 0.63, 95% CI: 0.45–0.89), male (HR = 0.63, 95% CI: 0.44–0.89), and obese participants with BMI ≥ 30 kg/m² (HR = 0.68, 95% CI: 0.48–0.95). Dose-response relationships within subgroups are visualized using restricted cubic splines in Supplementary Figure S3.

**Table 3:** Subgroup analysis of associations between dietary folate intake and MASLD based on baseline age, ethnicity, and BMI (model 4)

Characters	Participants/Case	Q2 (14512/164)		Q3 (14511/169)		Q4 (14512/146)		*P* for interaction	*P* for trend
		HR (95%CI)	*P*	HR (95%CI)	*P*	HR (95%CI)	*P*		
Age								0.299	
< 60years	33,903/384	0.77(0.58–1.01)	0.062	0.93(0.70–1.23)	0.598	0.63(0.45–0.89)	**0.008**		**0.021**
≥ 60years	24,144/307	1.01(0.73–1.40)	0.936	0.92(0.66–1.30)	0.650	0.96(0.66–1.39)	0.826		0.618
Sex								0.912	
Male	26,931/335	0.79(0.58–1.07)	0.128	0.77(0.56–1.05)	0.099	0.63(0.44–0.89)	**0.010**		**0.012**
Female	31,116/356	0.93(0.70–1.24)	0.610	1.07(0.80–1.44)	0.653	0.89(0.63–1.26)	0.518		0.678
BMI								0.456	
< 25 kg/m^2^	20,973/85	1.06(0.60–1.89)	0.832	0.99(0.53–1.86)	0.979	0.62(0.29–1.35)	0.229		0.254
25–30 kg/m^2^	24,374/228	0.86(0.59–1.26)	0.445	0.86(0.59–1.27)	0.454	0.96(0.63–1.45)	0.831		0.992
≥ 30 kg/m^2^	12,700/378	0.79(0.59–1.05)	0.102	0.91(0.68–1.21)	0.504	0.68(0.48–0.95)	**0.024**		**0.030**

BMI: Body mass index. Model 4: adjusted for age, sex, ethnicity, BMI, education level, TDI, physical activity, energy intake, smoking status, alcoholstatus, hypertension, and diabetes. Q1: the reference group. HR: hazard ratio. CI: confidence interval. P: significant level; The P was in bold to indicate statistical significance.

### Sensitive analysis

To test the stability of primary results, a number of sensitivity analyses were adopted. Overall, the negative association between highest dietary folate intake with MASLD were almost consistent with the results of cohort study, which reflected the stability of our findings (Supplementary Tables S2 - S10). Specifically, (1) We excluded participants with ≤ 2 years of follow-up time. The results of this sensitive analysis were in accordance with the results of cohort study. (Supplementary Table S2). (2) We removed participants with extreme energy intake. After the analysis, the dietary folate intake still significantly reduced the risk of MASLD (Supplementary Table S3). (3) We excluded participants with extreme dietary folate intake. In this analysis, the significant protective effect of Q4 still existed (Supplementary Table S4). (4) We excluded participants who took folate or multivitamin supplements. The analysis results supported that dietary folate intake had a stable inverse association with MASLD (Supplementary Table S5). (5) We excluded participants who had hypertension or diabetes at baseline. In this sensitive analysis, its analysis results supported our primary results (Supplementary Table S6). (6) After excluding participants with other liver diseases at baseline, the analysis results remained stable (Supplementary Table S7). (7) We used WC to replace BMI for the sensitivity analysis, and the results were basically in keeping with the main effects (Supplementary Table S8). (8) After adjusting energy intake for folate using the residual method, the results remained consistent with our primary finding (Supplementary Table S9). (9) After using the energy-standardized average value of folate and adjusting for dietary fiber and consumption of fruits and vegetables, the observed associations remained stable (Supplementary Table S10).

## Discussion

The results from this large-scale cohort research provided evidence of a substantial inverse association between dietary folate intake with MASLD. Participants in Q4 group had a 24% lower risk of developing MASLD relative to Q1 group. This relationship was characterized by a non-linear, L-shaped dose-response relationship, whereby the protective effect was most pronounced at lower intake levels and plateaued beyond approximately 300 µg. This association remained consistent with the results of cohort study across various subgroups and was robust throughout a series of rigorous sensitivity analyses. Furthermore, mediation analysis indicated that higher dietary folate could decrease the risk of MASLD by reducing the level of CRP.

Although the association between dietary folate intake with the newly defined MASLD was not probed in previous studies, our findings consistent with most existing evidence regarding the link between folate and NAFLD. For instance, previous cross-sectional studies had discovered significantly lower serum folate concentrations in patients with NAFLD compared to healthy controls [43]. More recently, studies in American adults also found an negative association between dietary folate intake with NAFLD [24, 44]. These findings were also supported by animal models. An animal study had shown that folate deficiency could exacerbate hepatic steatosis and inflammation, potentially through mechanisms involving impaired one-carbon metabolism and increased oxidative stress [45].

MASLD had a highly complex pathogenesis, which involved interactions among metabolic, inflammatory, genetic, and environmental factors [46]. Folate may confer protection through several interconnected pathways. Primarily, folate is a critical coenzyme in one-carbon metabolism and is crucial for the remethylation of homocysteine (Hcy) to methionine [47, 48]. Inadequate folate intake can impair this pathway, leading to Hyperhomocysteinemia (HHcy) [49]. Elevated Hcy levels linked to pro-inflammatory effects and insulin resistance, both central drivers of MASLD pathogenesis [50]. Our mediation analysis provided strength evidence for this anti-inflammatory pathway, demonstrating that the protective effect of dietary folate against MASLD was partially achieved by reducing CRP levels, accounting for approximately 6.56% of the total effect. Furthermore, folate is also essential for the synthesis of S-adenosylmethionine (SAM) [51]. SAM is a common methyl donor, and its depletion has been directly linked to liver injury, steatosis, and even fibrosis [52]. Therefore, adequate dietary folate may protect against MASLD by maintaining one-carbon metabolism and ensuring adequate SAM production [51].

Stratified analyses revealed that the inverse association between dietary folate intake and MASLD was more pronounced in specific subgroups, including individuals aged < 60 years, male, and those with obesity (BMI ≥ 30 kg/m²). The differences among these subgroups may be attributable to distinct pathophysiological characteristics. For example, studies have shown that the elderly exhibit significantly reduced nutrient absorption capacity, including calcium, iron, zinc, and vitamins, due to a decline in gastrointestinal function, which may attenuate the protective effect of folate and suggest that higher folate intake may be required in the elderly to meet demands [53–57]. Additionally, obesity is characterized by a state of chronic low-grade inflammation and oxidative stress. Accumulating evidence indicates that obese individuals are more susceptible to folate deficiency due to elevated inflammatory and oxidative stress burdens, which may increase their demand for folate [58–61]. As such, the protective effect of folate may be more pronounced in this population. Regarding sex differences, we observed that dietary folate intake was significantly higher in male (median [IQR]: 322.57[167.29]µg) than in female (median [IQR]: 291.11[151.18]µg) (*P* < 0.001). This observation may partly explain the more pronounced protective effect of folate in male; however, further large-scale studies are warranted to explore the underlying mechanisms. Collectively, these findings suggest that the protective role of folate may be modified by individual characteristics, highlighting the importance of personalized nutritional strategies in MASLD prevention.

Our research has several notable strengths: First, this was the novel investigation of dietary folate with MASLD under its new definition, and concluded that higher dietary folate intake could reduce the risk of MASLD. Second, our study elucidated the dose-response relationship and explored the mediating effects of inflammatory biomarker CRP, which add depth to our findings. Third, stratified analyses and sensitivity analyses provided strong support for the stability of our results. Finally, our research is a large-scale, population-based design. The comprehensive data allowed us to adjust for a wide array of potential confounding, making our results more persuasive.

Nonetheless, several limitations must be acknowledged. First, dietary folate intake was estimated through a 24-hour dietary questionnaire, which was susceptible to recall bias. In addition, the questionnaire may not reflect long-term dietary patterns, which may potentially lead to bias of the true association. Second, as an observational study, we couldn’t completely rule out reverse causality, and the potential for residual confounding from unmeasured variables remains. Third, the diagnosis of MASLD was relayed on ICD-10 code, rather than systematic imaging or biopsy, which may lead to misdiagnosis or missed diagnosis of the outcome. Fourth, the study cohort was predominantly European ancestry, which may affect the applicability of our results to other populations with different ethnic backgrounds, dietary habits, and MASLD risk profiles. Finally, due to data limitations, this study did not analyze serum/red blood cell folate and homocysteine biomarkers in the UK Biobank. Future studies should combine dietary intake and biochemical markers to more accurately assess folate status and its association with MASLD.

## Conclusions

This research demonstrated a significantly inverse association between dietary folate intake and risk of MASLD. This protective effect is partly mediated by reducing the level of CRP and shows an L-shaped nonlinear relationship. In the field of public health, our results suggested that promoting the consumption of folate-rich foods could be a viable, low-cost dietary strategy contributing to the primary prevention of MASLD. Future researches are warranted to confirm the causal role of folate in MASLD prevention.

## Electronic Supplementary Material

Below is the link to the electronic supplementary material.

Supplementary material 1.

Supplementary material 2.

## Data Availability

Publicly available datasets were analyzed in this study. This data can be found here: the UK Biobank (https://www.ukbiobank.ac.uk/).

## Abbreviations

BMI: Body mass index
C-Index: Concordance index
CI: Confidence interval
CRP: C-reactive protein
Hcy: Homocysteine
HHcy: Hyperhomocysteinemia
HR: Hazard ratio
ICD-10: International Classification of Diseases, Tenth Revision
IQR: Interquartile range
MASLD: Metabolic dysfunction-associated steatotic liver disease
NAFLD: Non-alcoholic Fatty disease
RBC: Red blood cells
RCS: Restricted cubic splines
SAM: S-adenosylmethionine
TDI: Townsend Deprivation Index
UKB: UK Biobank
WC: Waist circumference
