# Supplementary material for: Dietary folate intake and metabolic dysfunction-associated steatotic liver disease: a prospective cohort study
Source: Nutr Metab (Lond). 2026 May 20;23:85. doi: 10.1186/s12986-026-01141-0 (PMC13366692; doi:10.1186/s12986-026-01141-0)
Supplement: Supplementary file 1 — Supplementary material 1. [file 12986_2026_1141_MOESM1_ESM.docx]

**Dietary folate intake and Metabolic dysfunction-associated steatotic liver disease: a prospective cohort study**

**Supplementary Tables:**

**Table S1.** The UKB codes of the variables used in the paper

**Table S2.** Longitudinal associations between dietary folate intake and MASLD after excluding participants with ≤ 2 years of follow-up (N=57733)

**Table S3.** Longitudinal associations between dietary folate intake and MASLD after excluding participants with extreme energy intake (N=56883)

**Table S4.** Longitudinal associations between dietary folate intake and MASLD after excluding participants with extreme dietary folate intake (N=56881)

**Table S5.** Longitudinal associations between dietary folate intake and MASLD after excluding participants with the use of folate or multivitamin supplements (N=52983)

**Table S6.** Longitudinal associations between dietary folate intake and MASLD after excluding participants with hypertension and diabetes (N=41327)

**Table S7.** Longitudinal associations between dietary folate intake and MASLD after excluding participants with other liver diseases at baseline (N=57872)

**Table S8.** Longitudinal associations between dietary folate intake and MASLD after adjusting for waist circumference (WC) rather than BMI (N=58032)

**Table S9.** Longitudinal associations between energy-standardized dietary folate intake and MASLD after using the residual method (N=58047)

**Table S10.** Longitudinal associations between energy-standardized average value of folate and MASLD after adjusting for dietary fiber, fruit, and vegetable intake (N=46180)

**Supplementary Figures:**

**Figure S1.** The results of proportion hazard assumption

**Figure S2.** Cumulative risk curves for MASLD according to quartiles of dietary folate intake

**Figure S3.** Restricted cubic spline plots of dietary folate intake and MASLD grouped by age, sex and BMI

**Table S1.** The UKB codes of the variables used in the paper

| Variables | Field number | Classification |
| --- | --- | --- |
| Folate | 26022 | Continuous |
| Age | 34 | Continuous |
| Sex | 31 | Female  male |
| Ethnicity | 21000 | White  No White |
| Body mass index (BMI) | 21001 | Continuous |
| Education level | 6138 | Below the college  College or above |
| Townsend deprivation index (TDI) | 22189 | Continuous |
| Physical activity  (according to International Physical Activity Questionnaire) | 22032 | Low  Mid  High |
| Energy intake | 26002 | Continuous |
| Smoke status | 20116 | Never  Previous  Current |
| Alcohol status | 20117 | Never  Previous  Current |
| Hypertension | 6150&41270 | Yes/NO |
| Diabetes | 41270 | Yes/NO |

**Table S2.** Longitudinal associations between dietary folate intake and MASLD after excluding participants with ≤ 2 years of follow-up (N=57733)

| Folate | Participants/Case | Model 1 | | Model 2 | | Model 3 | | Model 4 | |
| --- | --- | --- | --- | --- | --- | --- | --- | --- | --- |
|  |  | HR (95%CI) | *P* | HR (95%CI) | *P* | HR (95%CI) | *P* | HR (95%CI) | *P* |
| Q1 | 14434/201 | Ref | - | Ref | - | Ref | - | Ref | - |
| Q2 | 14433/155 | 0.77(0.62 - 0.95) | **0.013** | 0.83(0.67 - 1.02) | 0.074 | 0.85(0.69 - 1.06) | 0.148 | 0.85(0.69 - 1.06) | 0.147 |
| Q3 | 14433/155 | 0.77(0.62 - 0.95) | **0.014** | 0.83(0.68 - 1.03) | 0.094 | 0.88(0.70 - 1.10) | 0.249 | 0.88(0.70 - 1.10) | 0.250 |
| Q4 | 14433/135 | 0.67(0.54 - 0.84) | **<0.001** | 0.70(0.56 - 0.87) | **0.002** | 0.75(0.58 - 0.97) | **0.027** | 0.73(0.57 - 0.95) | **0.018** |
| *P* for trend | | **<0.001** | | **0.002** | | **0.039** | | **0.028** | |

HR: hazard ratio. CI: confidence interval. P: significant level. Q1: reference group. Model 1: not adjusted any covariates. Model 2: adjusted for age, sex, ethnicity, BMI, education level and TDI. Model 3: further adjusted for physical activity, energy intake, smoking status and alcohol status. Model 4: further adjusted for hypertension and diabetes.

**Table S3.** Longitudinal associations between dietary folate intake and MASLD after excluding participants with extreme energy intake (N=56883)

| Folate | Participants/Case | Model 1 | | Model 2 | | Model 3 | | Model 4 | |
| --- | --- | --- | --- | --- | --- | --- | --- | --- | --- |
|  |  | HR (95%CI) | *P* | HR (95%CI) | *P* | HR (95%CI) | *P* | HR (95%CI) | *P* |
| Q1 | 14222/205 | Ref | **-** | Ref | **-** | Ref | **-** | Ref | **-** |
| Q2 | 14220/160 | 0.78(0.63 - 0.95) | **0.016** | 0.83(0.68 - 1.03) | 0.088 | 0.85(0.69 - 1.05) | 0.137 | 0.85(0.69 - 1.06) | 0.144 |
| Q3 | 14220/167 | 0.81(0.66 - 0.99) | **0.048** | 0.88(0.72 - 1.09) | 0.238 | 0.91(0.73 - 1.13) | 0.382 | 0.91(0.73 - 1.13) | 0.390 |
| Q4 | 14221/140 | 0.68(0.55 - 0.85) | **<0.001** | 0.72(0.58 - 0.90) | **0.003** | 0.74(0.58 - 0.95) | **0.020** | 0.73(0.57 - 0.94) | **0.014** |
| *P* for trend | | **0.001** | | **0.007** | | **0.036** | | **0.026** | |

HR: hazard ratio. CI: confidence interval. P: significant level. Q1: reference group. Model 1: not adjusted any covariates. Model 2: adjusted for age, sex, ethnicity, BMI, education level and TDI. Model 3: further adjusted for physical activity, energy intake, smoking status and alcohol status. Model 4: further adjusted for hypertension and diabetes.

**Table S4.** Longitudinal associations between dietary folate intake and MASLD after excluding participants with extreme dietary folate intake (N=56881)

| Folate | Participants/Case | Model 1 | | Model 2 | | Model 3 | | Model 4 | |
| --- | --- | --- | --- | --- | --- | --- | --- | --- | --- |
|  |  | HR (95%CI) | *P* | HR (95%CI) | *P* | HR (95%CI) | *P* | HR (95%CI) | *P* |
| Q1 | 14221/198 | Ref | **-** | Ref | **-** | Ref | **-** | Ref | **-** |
| Q2 | 14220/161 | 0.81(0.66 - 0.99) | **0.046** | 0.87(0.70 - 1.07) | 0.185 | 0.89(0.72 - 1.10) | 0.273 | 0.89(0.72 - 1.10) | 0.288 |
| Q3 | 14220/167 | 0.84(0.69 - 1.03) | 0.102 | 0.91(0.74 - 1.12) | 0.389 | 0.94(0.76 - 1.17) | 0.585 | 0.94(0.76 - 1.17) | 0.605 |
| Q4 | 14220/139 | 0.70(0.57 - 0.87) | **0.001** | 0.74(0.59 - 0.92) | **0.007** | 0.77(0.60 - 0.99) | **0.039** | 0.76(0.59 - 0.97) | **0.028** |
| *P* for trend | | **0.003** | | **0.013** | | 0.065 | | **0.048** | |

HR: hazard ratio. CI: confidence interval. P: significant level. Q1: reference group. Model 1: not adjusted any covariates. Model 2: adjusted for age, sex, ethnicity, BMI, education level and TDI. Model 3: further adjusted for physical activity, energy intake, smoking status and alcohol status. Model 4: further adjusted for hypertension and diabetes.

**Table S5.** Longitudinal associations between dietary folate intake and MASLD after excluding participants with the use of folate or multivitamin supplements (N=52983)

| Folate | Participants/Case | Model 1 | | Model 2 | | Model 3 | | Model 4 | |
| --- | --- | --- | --- | --- | --- | --- | --- | --- | --- |
|  |  | HR (95%CI) | *P* | HR (95%CI) | *P* | HR (95%CI) | *P* | HR (95%CI) | *P* |
| Q1 | 13246/190 | Ref | **-** | Ref | **-** | Ref | **-** | Ref | **-** |
| Q2 | 13246/148 | 0.77(0.62 - 0.96) | **0.020** | 0.84(0.67 - 1.04) | 0.104 | 0.86(0.69 - 1.06) | 0.159 | 0.86(0.70 - 1.07) | 0.188 |
| Q3 | 13245/152 | 0.80(0.65 - 0.99) | **0.039** | 0.87(0.70 - 1.08) | 0.205 | 0.90(0.73 - 1.12) | 0.345 | 0.91(0.73 - 1.13) | 0.396 |
| Q4 | 13246/134 | 0.71(0.57 - 0.88) | **0.002** | 0.74(0.59 - 0.93) | **0.009** | 0.77(0.62 - 0.97) | **0.025** | 0.77(0.62 - 0.97) | **0.025** |
| *P* for trend | | **0.004** | | **0.015** | | 0.210 | | 0.170 | |

HR: hazard ratio. CI: confidence interval. P: significant level. Q1: reference group. Model 1: not adjusted any covariates. Model 2: adjusted for age, sex, ethnicity, BMI, education level and TDI. Model 3: further adjusted for physical activity, energy intake, smoking status and alcohol status. Model 4: further adjusted for hypertension and diabetes.

**Table S6.** Longitudinal associations between dietary folate intake and MASLD after excluding participants with hypertension and diabetes (N=41327)

| Folate | Participants/Case | Model 1 | | Model 2 | | Model 3 | |
| --- | --- | --- | --- | --- | --- | --- | --- |
|  |  | HR (95%CI) | *P* | HR (95%CI) | *P* | HR (95%CI) | *P* |
| Q1 | 10333/111 | Ref | **-** | Ref | **-** | Ref | **-** |
| Q2 | 10331/78 | 0.70(0.53 - 0.94) | **0.017** | 0.76(0.57 - 1.02) | 0.065 | 0.78(0.58 - 1.05) | 0.099 |
| Q3 | 10331/91 | 0.82(0.62 - 1.08) | 0.163 | 0.90(0.68 - 1.19) | 0.457 | 0.93(0.69 - 1.25) | 0.630 |
| Q4 | 10332/66 | 0.60(0.44 - 0.81) | **<0.001** | 0.65(0.48 - 0.89) | **0.007** | 0.67(0.47 - 0.96) | **0.028** |
| *P* for trend | | **0.003** | | **0.019** | | 0.068 | |

HR: hazard ratio. CI: confidence interval. P: significant level. Q1: reference group. Model 1: not adjusted any covariates. Model 2: adjusted for age, sex, ethnicity, BMI, education level and TDI. Model 3: further adjusted for physical activity, energy intake, smoking status and alcohol status.

**Table S7.** Longitudinal associations between dietary folate intake and MASLD after excluding participants with other liver diseases at baseline (N=57872)

| Folate | Participants/Case | Model 1 | | Model 2 | | Model 3 | | Model 4 | |
| --- | --- | --- | --- | --- | --- | --- | --- | --- | --- |
|  |  | HR (95%CI) | *P* | HR (95%CI) | *P* | HR (95%CI) | *P* | HR (95%CI) | *P* |
| Q1 | 14468/208 | Ref | **-** | Ref | **-** | Ref | **-** | Ref | **-** |
| Q2 | 14468/158 | 0.76(0.61 - 0.93) | **0.008** | 0.82(0.66 - 1.00) | 0.055 | 0.83(0.67 - 1.03) | 0.097 | 0.83(0.67 - 1.03) | 0.097 |
| Q3 | 14468/166 | 0.80(0.65 - 0.98) | **0.029** | 0.87(0.71 - 1.07) | 0.190 | 0.90(0.72 - 1.12) | 0.343 | 0.90(0.72 - 1.12) | 0.346 |
| Q4 | 14468/145 | 0.70(0.56 - 0.86) | **<0.001** | 0.73(0.59 - 0.91) | **0.005** | 0.76(0.60 - 0.98) | **0.035** | 0.75(0.59 - 0.96) | **0.024** |
| *P* for trend | | **0.002** | | **0.010** | | 0.063 | | **0.046** | |

HR: hazard ratio. CI: confidence interval. P: significant level. Q1: reference group. Model 1: not adjusted any covariates. Model 2: adjusted for age, sex, ethnicity, BMI, education level and TDI. Model 3: further adjusted for physical activity, energy intake, smoking status and alcohol status. Model 4: further adjusted for hypertension and diabetes.

**Table S8.** Longitudinal associations between dietary folate intake and MASLD after adjusting for waist circumference (WC) rather than BMI (N=58032)

| Folate | Participants/Case | Model 1 | | Model 2 | | Model 3 | | Model 4 | |
| --- | --- | --- | --- | --- | --- | --- | --- | --- | --- |
|  |  | HR (95%CI) | *P* | HR (95%CI) | *P* | HR (95%CI) | *P* | HR (95%CI) | *P* |
| Q1 | 14508/212 | Ref | **-** | Ref | **-** | Ref | **-** | Ref | **-** |
| Q2 | 14508/164 | 0.77(0.63 - 0.94) | **0.012** | 0.83(0.67 - 1.01) | 0.068 | 0.86(0.70 - 1.06) | 0.164 | 0.87(0.70 - 1.07) | 0.183 |
| Q3 | 14508/169 | 0.80(0.65 - 0.97) | **0.027** | 0.87(0.71 - 1.06) | 0.166 | 0.92(0.74 - 1.15) | 0.464 | 0.93(0.75 - 1.15) | 0.487 |
| Q4 | 14508/146 | 0.69(0.56 - 0.85) | **<0.001** | 0.71(0.58 - 0.88) | **0.002** | 0.78(0.61 – 1.00) | 0.054 | 0.77(0.60 - 0.99) | **0.040** |
| *P* for trend | | **0.001** | | **0.006** | | 0.062 | | **0.045** | |

HR: hazard ratio. CI: confidence interval. P: significant level. Q1: reference group. Model 1: not adjusted any covariates. Model 2: adjusted for age, sex, ethnicity, WC, education level and TDI. Model 3: further adjusted for physical activity, energy intake, smoking status and alcohol status. Model 4: further adjusted for hypertension and diabetes.

**Table S9.** Longitudinal associations between energy-standardized dietary folate intake and MASLD after using the residual method (N=58047)

| Folate | Participants/Case | Model 1 | | Model 2 | | Model 3 | | Model 4 | |
| --- | --- | --- | --- | --- | --- | --- | --- | --- | --- |
|  |  | HR (95%CI) | *P* | HR (95%CI) | *P* | HR (95%CI) | *P* | HR (95%CI) | *P* |
| Q1 | 14512/210 | Ref | **-** | Ref | **-** | Ref | **-** | Ref | **-** |
| Q2 | 14512/177 | 0.84(0.69 - 1.02) | 0.085 | 0.85(0.70 - 1.04) | 0.123 | 0.85(0.70 - 1.04) | 0.116 | 0.85(0.70 - 1.04) | 0.120 |
| Q3 | 14511/148 | 0.70(0.57 - 0.87) | **<0.001** | 0.70(0.57 - 0.86) | **<0.001** | 0.70(0.57 - 0.86) | **<0.001** | 0.71(0.58 - 0.88) | **0.002** |
| Q4 | 14512/156 | 0.74(0.60 - 0.91) | **0.004** | 0.73(0.59 - 0.90) | **0.003** | 0.73(0.59 - 0.89) | **0.002** | 0.74(0.60 - 0.90) | **0.004** |
| *P* for trend | | **0.002** | | **0.001** | | **<0.001** | | **0.002** | |

HR: hazard ratio. CI: confidence interval. P: significant level. Q1: reference group. Model 1: not adjusted any covariates. Model 2: adjusted for age, sex, ethnicity, BMI, education level and TDI. Model 3: further adjusted for physical activity, smoking status and alcohol status. Model 4: further adjusted for hypertension and diabetes.

**Table S10.** Longitudinal associations between energy-standardized average value of folate and MASLD after adjusting for dietary fiber, fruit, and vegetable intake (N=46180)

| Folate | Participants/Case | Model 1 | | Model 2 | | Model 3 | | Model 4 | |
| --- | --- | --- | --- | --- | --- | --- | --- | --- | --- |
|  |  | HR (95%CI) | *P* | HR (95%CI) | *P* | HR (95%CI) | *P* | HR (95%CI) | *P* |
| Q1 | 11545/151 | Ref | **-** | Ref | **-** | Ref | **-** | Ref | **-** |
| Q2 | 11545/117 | 0.77(0.61 - 0.98) | **0.035** | 0.78(0.61 – 0.99) | **0.042** | 0.79(0.62 - 1.01) | 0.063 | 0.80(0.63 - 1.02) | 0.067 |
| Q3 | 11545/129 | 0.85(0.67 – 1.08) | 0.181 | 0.85(0.67 – 1.08) | 0.188 | 0.89(0.70 – 1.13) | 0.348 | 0.92(0.72 – 1.16) | 0.473 |
| Q4 | 11545/108 | 0.71(0.56 – 0.91) | **0.007** | 0.70(0.55 – 0.90) | **0.005** | 0.75(0.58 – 0.97) | **0.030** | 0.77(0.60 – 0.99) | **0.045** |
| *P* for trend | | **0.017** | | **0.012** | | 0.062 | | 0.097 | |

HR: hazard ratio. CI: confidence interval. P: significant level. Q1: reference group. Model 1: not adjusted any covariates. Model 2: adjusted for age, sex, ethnicity, BMI, education level and TDI. Model 3: further adjusted for physical activity, dietary fiber, fruit, vegetable, smoking status and alcohol status. Model 4: further adjusted for hypertension and diabetes.


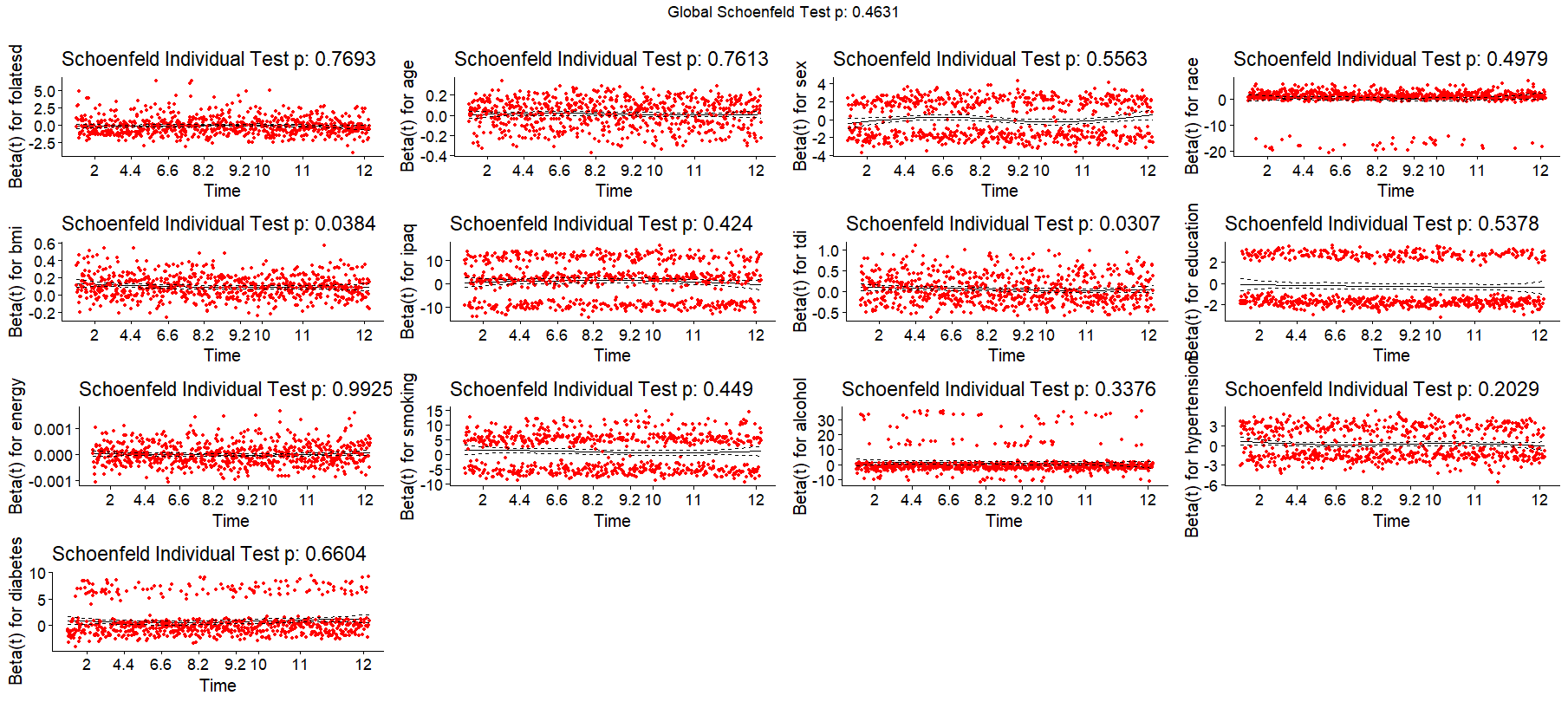


**Figure S1.** The results of proportion hazard assumption. The *P*-values of the global Schoenfeld test results and dietary folate intake were 0.4631 and 0.7693, which can be considered this Cox regression model met the proportional hazards assumption


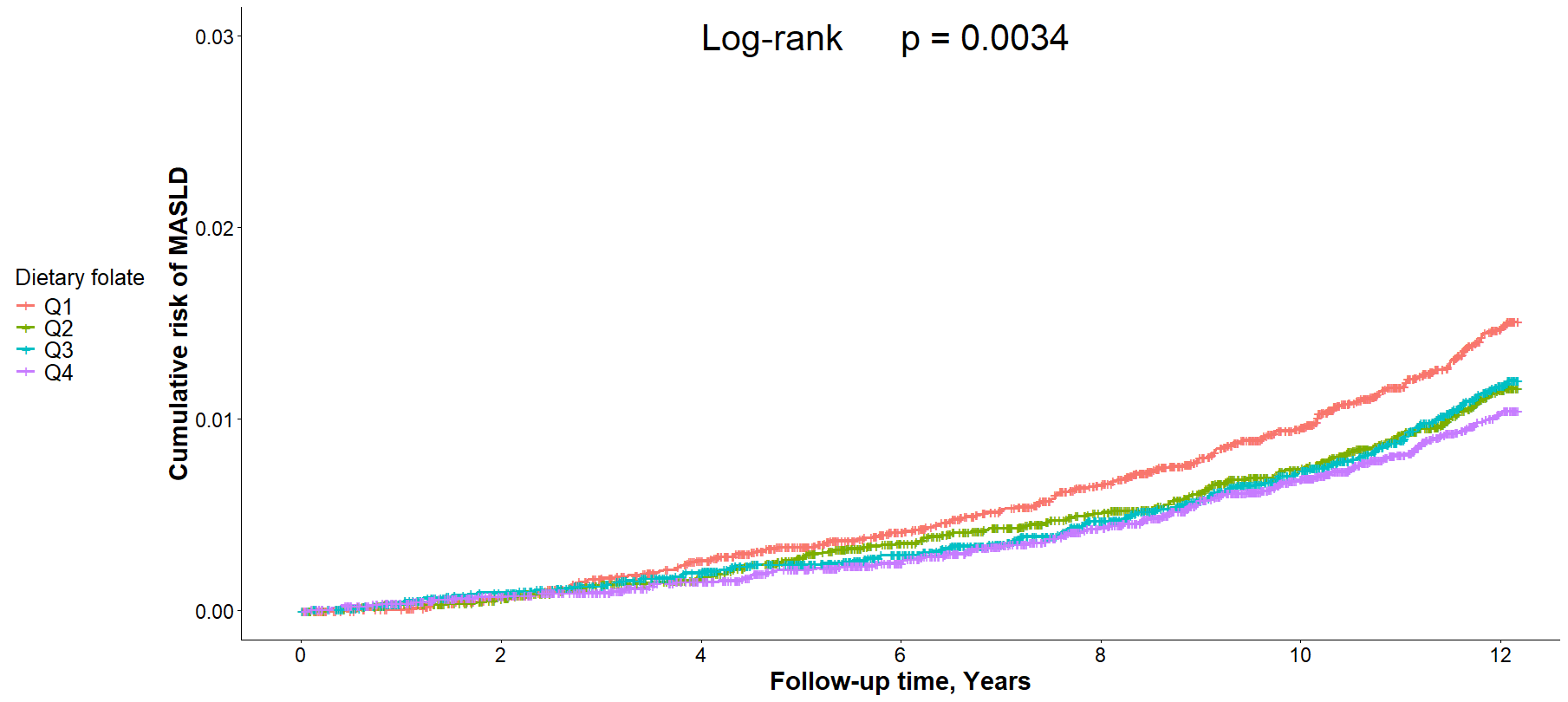


**Figure S2.** Cumulative risk curves for MASLD according to quartiles of dietary folate intake. Q1, Q2, Q3, and Q4 are calculated based on the quartiles of dietary folate.


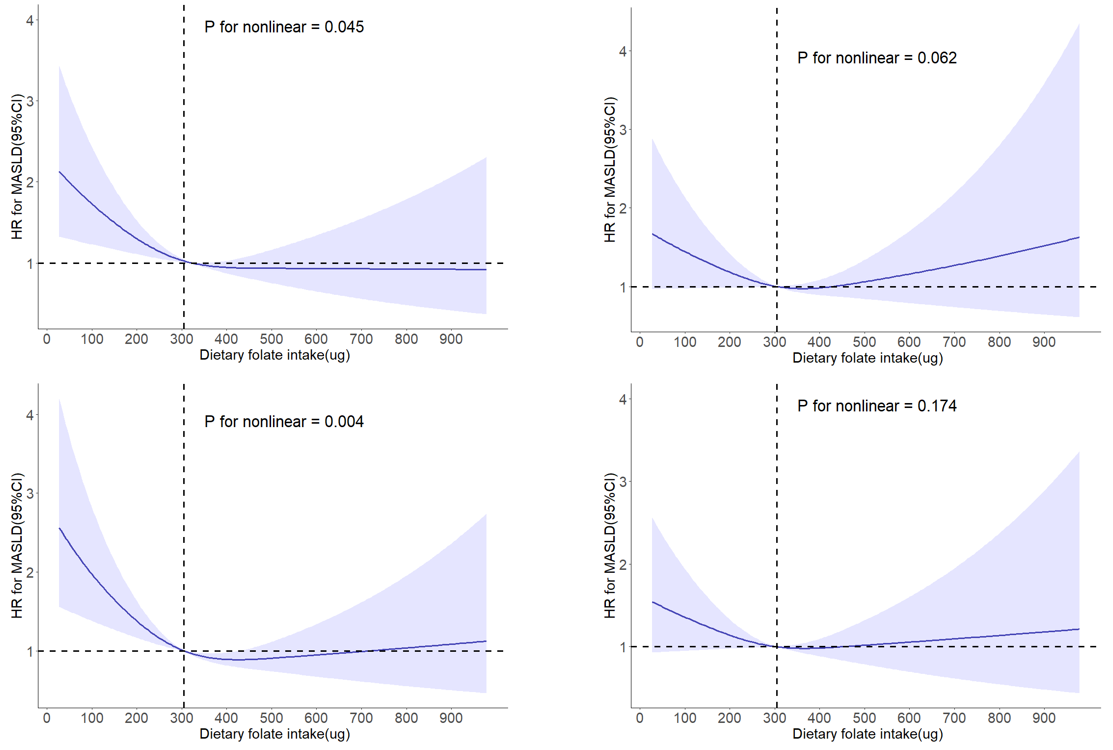


(a)

(b)

(c)

(d)


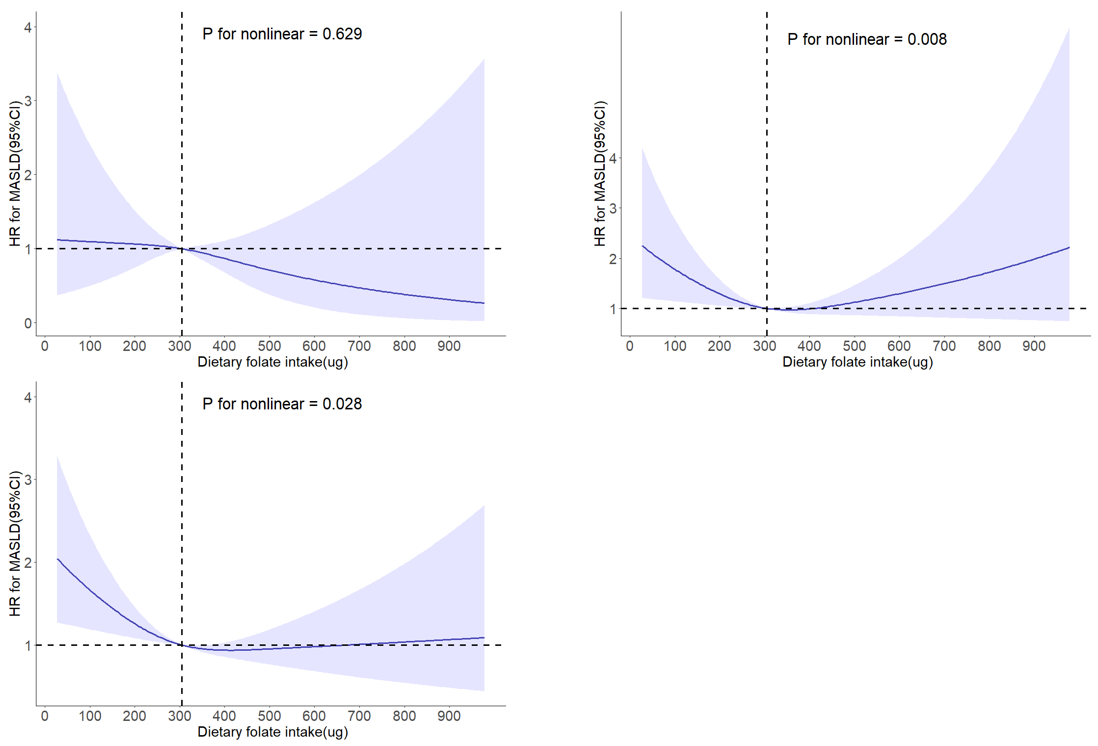


(g)

(e)

(f)

**Figure S3.** Restricted cubic spline plots of dietary folate intake and MASLD grouped by age, sex and BMI. Note: From left to right and from top to bottom, it is in the following order: (a): < 60 years, (b): ≥ 60 years, (c): male, (d): female, (e): normal (<25 kg/m^2^), (f): overweight (25-30 kg/m^2^), (g): fat (≥30 kg/m^2^). The blue lines are HR estimates and the light blue areas are 95%CI for HR. HR: hazard ratios; CI: confidence intervals.
