# Supplementary figures and images for: Dietary folate intake and metabolic dysfunction-associated steatotic liver disease: a prospective cohort study
Source: Nutr Metab (Lond). 2026 May 20;23:85. doi: 10.1186/s12986-026-01141-0 (PMC13366692; doi:10.1186/s12986-026-01141-0)

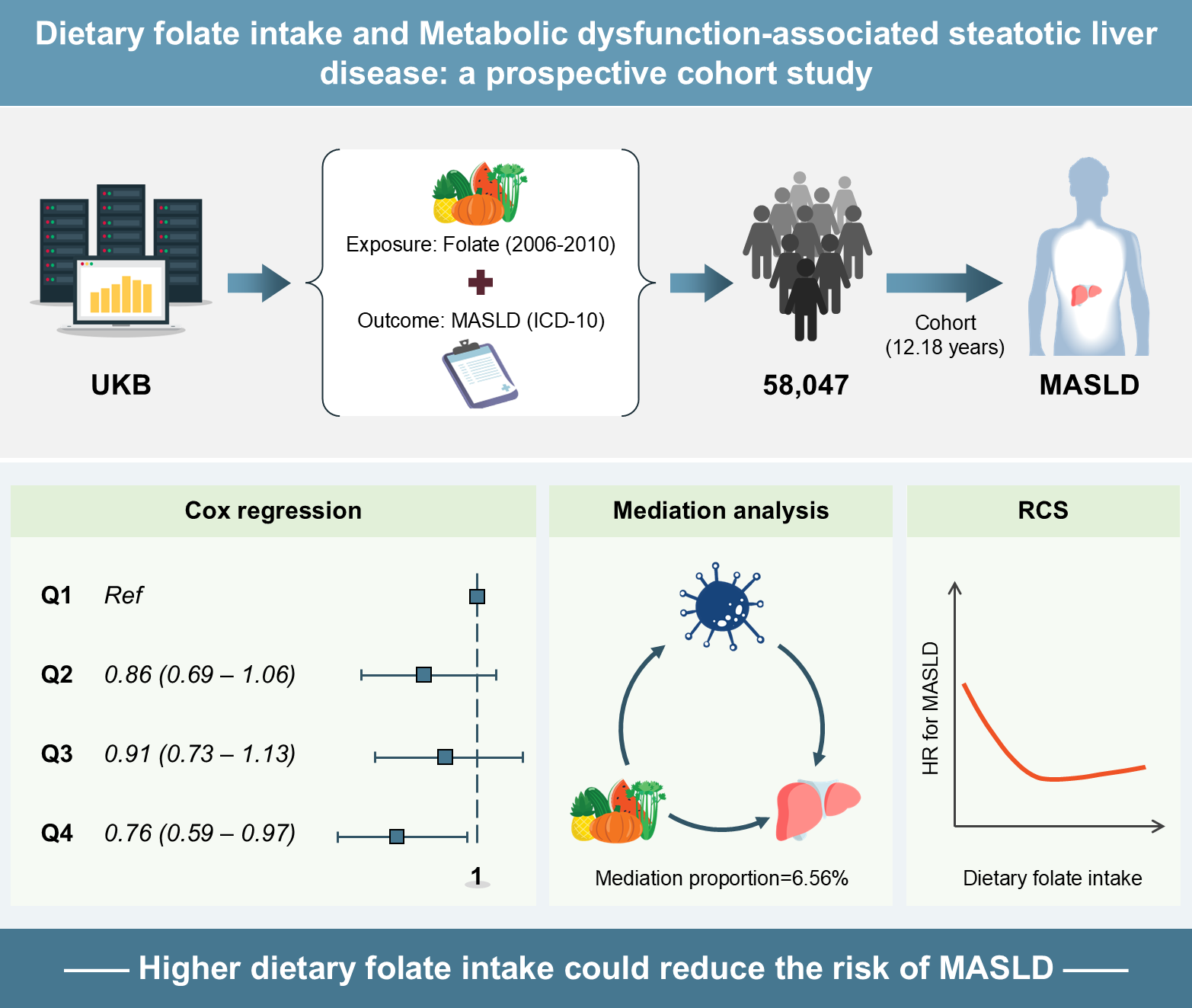

Supplement: Supplementary file 2 — Supplementary material 2. [file 12986_2026_1141_MOESM2_ESM.png]
